# Supplementary material for: Portrait of Molecular Signaling and Putative Therapeutic Targets in Prostate Cancer with ETV4 Fusion
Source: Biomedicines. 2022 Oct 20;10(10):2650. doi: 10.3390/biomedicines10102650 (PMC9599949; doi:10.3390/biomedicines10102650)
Supplement: Supplementary file 1 [file biomedicines-10-02650-s001.zip › Supplementary data.pdf]

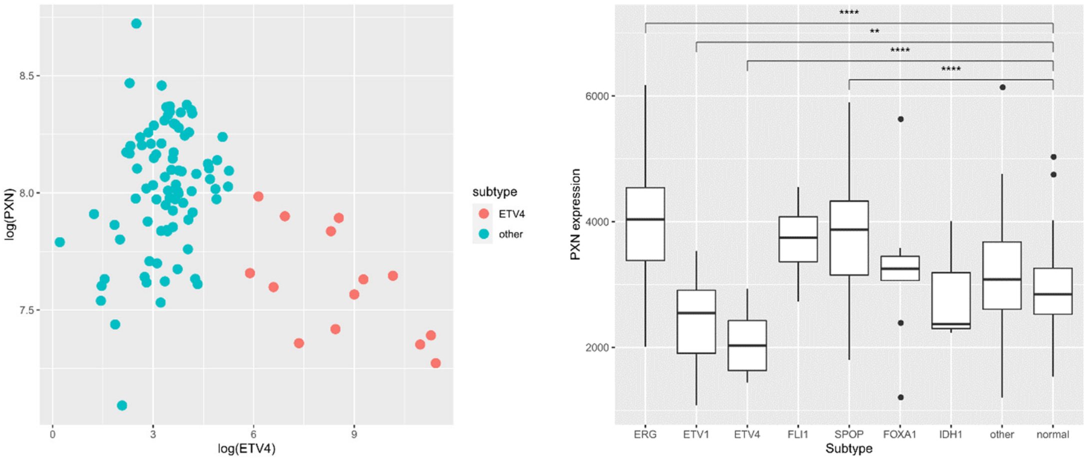

(**a**) (**b**)

Figure S1. Pattern of PXN expression in prostate cancer: (**a**) Dot plot of PXN expression and ETV4 expression. The x-axis is the log value of ETV4 expression level and the y-axis is the log value of PXN expression; (**b**) Boc plot of PXN expression in prostate cancer according to subtype. Classification of each subtype was based on a prior study and normal group samples were from the TCGA database with the sample code 11.**$p \leq 0.01$ ****$p \leq 0.0001$.

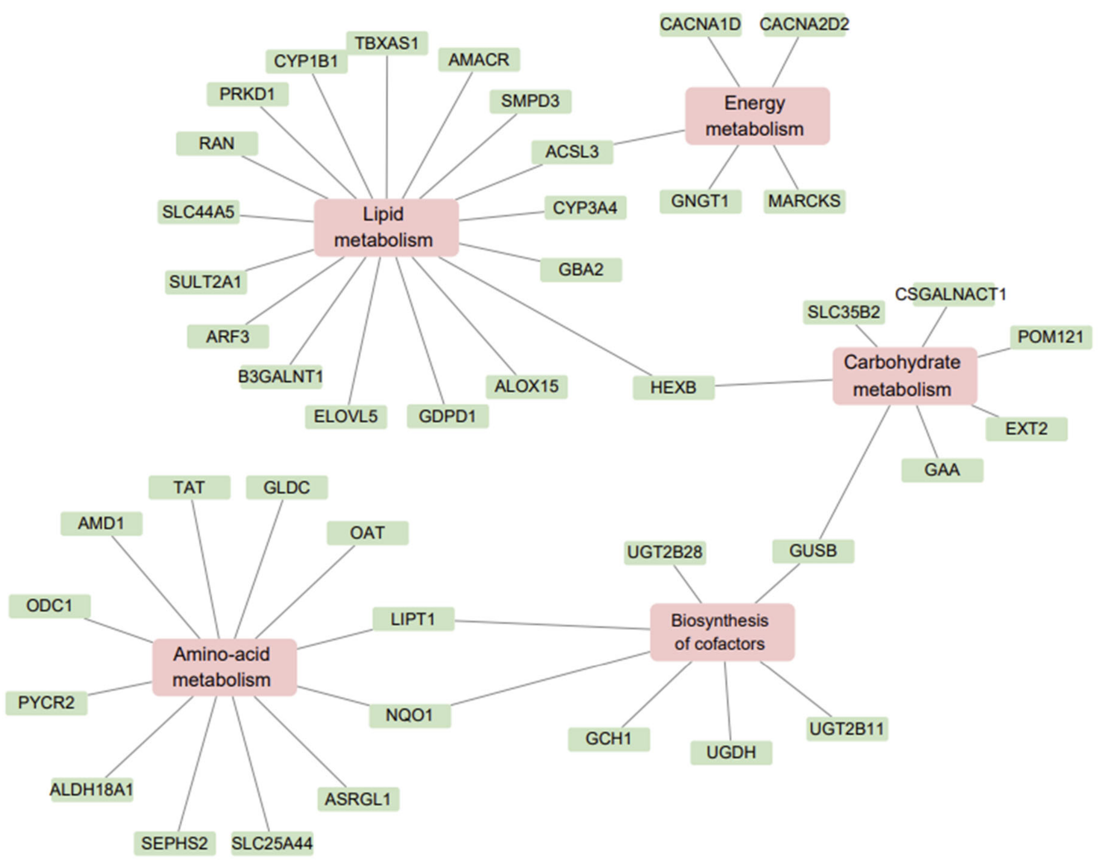

Figure S2. Gene-pathway network about metabolism pathway. Red round rectangles are major pathways related to metabolism. Green round rectangles are genes.
